# Elevated Temperature, Nitrate and Diesel Oil Enhance the Distribution of the Opportunistic Pathogens *Scedosporium* spp.

**DOI:** 10.3390/jof9040403

**Published:** 2023-03-24

**Authors:** Johannes Rainer, Marlene Eggertsberger

**Affiliations:** 1 Institute of Microbiology, Universität Innsbruck, Technikerstraße 25, 6020 Innsbruck, Austria; 2 Independent Researcher, Via Livizzani 44, 41121 Modena, Italy

**Keywords:** *Scedosporium*, diesel, nitrate, temperature, population, infection route, reservoir

## Abstract

*Scedosporium* infections mainly occur after aspiration of contaminated water or inoculation with polluted environmental materials. *Scedosporium* spp. have been isolated from anthropogenic environments frequently. To understand their propagation and routes of infection, possible reservoirs of *Scedosporium* spp. should be explored. In this study, the impact of temperature, diesel and nitrate on *Scedosporium* populations in soil is described. Soil was treated with diesel and KNO_3_ and incubated for nine weeks at 18 and 25 °C. Isolation of *Scedosporium* strains was done using SceSel+. For the identification of 600 isolated strains, RFLP and rDNA sequencing were used. *Scedosporium apiospermum*, *S. aurantiacum*, *S. boydii* and *S. dehoogii* were isolated at the beginning and/or the end of incubation. Temperature alone had a minor effect on the *Scedosporium* population. The combination of 25 °C and nitrate resulted in higher *Scedosporium* numbers. Treatment with 10 g diesel/kg soil and incubation at 25 °C resulted in even higher abundance, and favored *S. apiospermum* and *S. dehoogii*. The results of this study show that diesel-polluted soils favor dispersal of *Scedosporium* strains, especially *S. apiospermum* and *S. dehoogii.* Higher temperature force the effect of supplementations.

## 1. Introduction

The Microascaceae (Microascales, Ascomycota) contain predominantly saprobic fungi and opportunistic human pathogens. Representatives of this family belonging to the genus *Scedosporium* are widely known as causative agents of cutaneous infections [1], lung colonization in patients with cystic fibrosis (CF) [2] and various deep infections [3,4,5]. They have been shown to possess a partly intrinsic ability of immune evasion [6]. *S. apiospermum* was previously described as the most abundant agent of *Scedosporium* infections [7]. With emerging reports of infections, it is of great importance to gain better knowledge of the abundance and ecological niche of *Scedosporium*. 

As soil is one of the main infectious agents, investigations of abundance and distribution of *Scedosporium* in the environment were previously carried out [8,9,10]. Strains have been isolated from a wide range of environments, mainly from anthropogenic habitats [11], oil-soaked soils, cattle dung and sewage [12]. April et al. [12] and Kaltseis et al. [8] suggested a natural occurrence in nutrient-rich or polluted environments, such as over-fertilized agricultural soils or oil-contaminated sites.

Kaltseis et al. [8] compared species distribution in the environment with the distribution observed in clinical cases. They showed that *S*. *boydii* caused most infections with involvement of the central nervous system (CNS), whereas this fungus was less abundant in soils. *Scedosporium apiospermum*, however, was involved in a large proportion of clinical cases and frequently isolated from soils. This was also shown in studies from other geographical regions in North Africa and Asia [10,13]. *Scedosporium aurantiacum* was not abundant in soils at least in middle Europe and rarely isolated from clinical sources. In contrast, *S. dehoogii* caused only isolated infections, but was frequently isolated from soils.

*Scedosporium* spp. can grow at high temperatures up to 40 °C. This feature enables *Scedosporium* species to survive in humans. In a preliminary, unpublished survey concerning soil temperatures in cities up to 31 °C in urban soils in 15 cm depth were measured. Another relevant factor for the environmental distribution is the availability of nitrogen compounds. Zacke et al. [14] demonstrated strain selections through different concentrations of nitrate in culture. Furthermore, Ulfig et al. [15] and Kaltseis et al. [8] found a positive correlation between ammonium concentration and frequency of *Scedosporium*. Nitrate, which can be used by organisms directly, is readily soluble and easy to leach. The nitrogen concentration in soils is affected by climate, vegetation- and soil characteristics. In agricultural soils and industrial areas, fertilization and cultivation play an important role concerning nitrogen content. Ammonium (NH_4_) is the second major source of nitrogen compounds in soils and is mainly due to agricultural emission [16].

The second tested supplement besides different nitrogen concentrations was diesel oil, a characteristic industrial pollutant present in urban environments. Ulfig et al. [9] reported that some *Scedosporium* isolates assimilate oil alkanes. Among the tested substrates (diesel, biodiesel, rapeseed oil), diesel oil hydrocarbons were found to be the most resistant to biodegradation in general. Claußen & Schmidt [17] demonstrated that *S. apiospermum* has the ability to degrade phenol and p-cresol for the use as carbon and energy source. Morales et al. [18] found a wide range of genes in *S. apiospermum* involved in the degradation of hydrocarbons like halogenated alkanes. 

Kaltseis et al. [8] found up to 7.000 CFU/g *Scedosporium* spp. in soils near petrol stations, and near highly frequented roads, whereas from 100 other sampling sites in natural habitats in Austria and The Netherlands, no strains could be isolated. Findings of *S. dehoogii* increased with the degree of human impact. *S. apiospermum* was isolated mainly from agricultural soils, parks, and playgrounds. *S. boydii* and *S. aurantiacum* were less abundant. *S. minutisporum* was isolated only from soil in industrial areas. As many hydrocarbons produced from mineral oil are presumably poisonous to many organisms, described abilities can work as selective features for *Scedosporium* populations while colonising man-made habitats. These may serve as reservoirs for infecting populations.

An appropriate definition of the ecological niche of *Scedosporium* species is of high epidemiologic interest to evaluate the infection risks for people in different environments. This is the first study on a possible effect of soil pollution with diesel and nitrate on natural populations of *Scedosporium* under controlled conditions. Its aim is to investigate the shift in *Scedosporium* populations affected by temperature, diesel, and nitrogen.

## 2. Materials and Methods

Soil was taken from a lawn in front of a university building in Innsbruck. The sampling depth was up to 30 cm. After removing stones and roots, soil was filled into ten glass containers, approximately 6 kg each, equipped with a top cover with grinding. Soil samples were supplemented with different concentrations of diesel (5 and 10 g/kg soil) or nitrogen (KNO_3_: 2 and 4% *w*/*v*) in two parallels for each supplement. These values were chosen on the basis of legal thresholds and practical implications. One series of parallels was incubated at 18 °C, the other at 25 °C. Untreated controls were included for each temperature. Containers were sampled for fungi every three weeks: A total of 100 g soil was taken from each glass at five different spots in every single container and mixed manually. From the mixed samples 3–5 g soil were used for the isolation of *Scedosporium* strains.

For the isolation, each of three sample aliquots was diluted 1:10 under aseptic conditions with autoclaved Tween^®^80 0.001%/NaCl 0.85% (*w*/*v*). Soil samples were suspended and extracted in 50 mL screw cap tubes on a head-over-head-shaker for 1 h at room temperature. The extract solution was diluted in a decimal dilution series up to step 10^−5^. From every single dilution eight SceSel+ [19] parallel plates were inoculated with 300 µL or 400 µL (differed for technical reasons) of the extract. SceSel+-plates were incubated at 37 °C for one week. Colony numbers were referred to soil dry weight (dw). Dry weight was measured with 5 g soil of each sample. For analysis, the mean values (Mean), medians (Med), standard deviations (s), minimum- and maximum CFU per gram soil (dw) of *Scedosporium* strains were calculated (data provided as Supplementary Materials) as well as *p* values (paired *t*-test two tail) for all treatments.

All isolates derived (2321) were controlled through their cultural and morphological characteristics. To assure the correct identification of the different *Scedosporium* species, RFLP digestion-profiles of most strains were performed and compared. These were used for further analysis. Banding patterns showed varying sizes and numbers of fragments (Table 1) for each species. The digestion based on ITS region was carried out following Rainer et al. [4] with AluI, HaeIII, HinfI, RsaI and Sau3AI. A set of ten strains of each RFLP banding pattern was chosen randomly and underwent ITS-sequencing with primers ITS5/ITS4 (ITS5: 5′-ggAAgTAAAAgTCgTAACAAgg-3′, ITS4: 5′-TCCTCCgCTTATTgATATgC-3′) [20] for further evaluation and standardization of the RFLP-profiles. The assignment of sequences to certain species was based on the curated database Mycobank. The qualitative and quantitative analysis of the *Scedosporium* population was traced by characterizing the strains on species level from the samples at week 0 and week 9. 

## 3. Results

**Figure 1:**
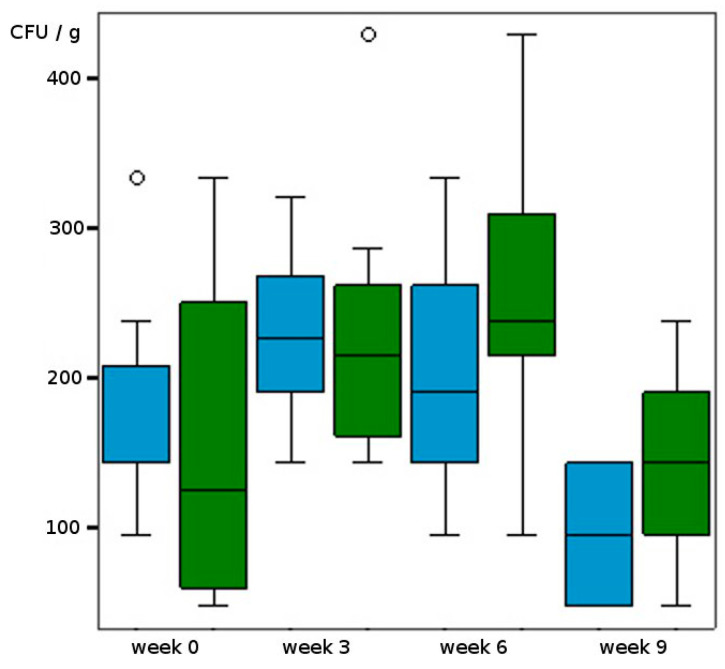
Alteration of CFU/g soil (dw) at different incubation temperatures (blue: 18 °C, green: 25 °C, circles: outliers).

**Figure 2:**
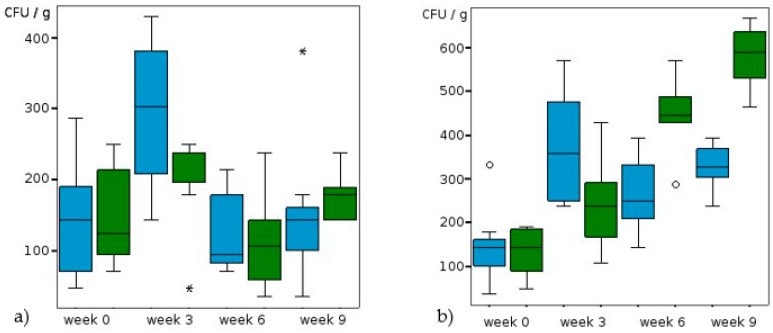
Alteration of CFU/g soil (dw) at (**a**) 18 °C and (**b**) 25 °C and different KNO3 concentrations (blue: 2%, green: 4%, stars: extreme values, circles: outliers).

**Figure 3:**
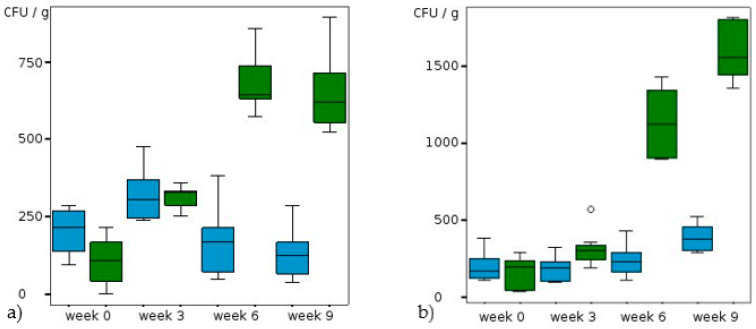
Alteration of CFU/g soil (dw) at (**a**) 18 °C and (**b**) 25 °C and different diesel concentrations (blue: 5 g/kg soil, green: 10 g/kg soil, circles: outliers).

Comparison of the untreated reference trials showed a barely detectable effect of temperature on the *Scedosporium* population (Table 2 and Table 3, Figure 1). In the references the highest concentration of *Scedosporium* strains was found after 6 weeks of incubation at 25 °C. At that time a mean value of 238 CFU/g soil (dw) was isolated. After that, populations decreased to mean concentrations of 95 CFU/g (18 °C) and to 143 CFU/g, respectively (25 °C) (Figure 1). Furthermore, KNO_3_ treatment at 18 °C did not lead to a clear shift of the population. In contrast there was a clear tendency towards higher abundance of *Scedosporium* spp. at 25 °C and higher nitrate concentration with mean CFU values of up to 580 CFU/g after 9 weeks (*p* < 0,001; Table 3, Figure 2a,b). However also with 2% KNO_3_ the increase of the population was significant (*p* = 0,001) at 25 °C. The most pronounced effect was found in the trials with diesel treatment (Table 2 and Table 3, Figure 3a,b). At 18 °C and with 10 g diesel/kg soil the abundance rose from 106 to 649 CFU/g mean values in 9 weeks (*p* ˂ 0.001). At 25 °C and with 10 g diesel/kg, the mean *Scedosporium* counts rose tenfold, starting from 159 to 1594 CFU/g (dw) during the observation period (*p* < 0.001). For 5 g diesel/kg the effect was hardly detectable at 18 °C. 

The isolated *Scedosporium* strains represented four species: *S. apiospermum*, *S. aurantiacum*, *S. boydii* and *S. dehoogii*. In Table 1 the diagnostic RFLP-matrix used for comparative analyses is shown. After sequencing the ITS-regions of selected strains, it was possible to identify all species only by means of RFLP. This method was therefore found to be a suitable identification tool for this study.

Focusing on species distribution, a shift within the observed population of *Scedosporium* species were traced over nine weeks. The proportion of isolated *S. apiospermum* strains decreased at both 18 and 25 °C after nine weeks in untreated soil. In contrast, strains of other three detected species showed no apparent quantitative alteration at the different growth temperatures in untreated soil (Table 2 and Table 3).

Exposure to 2% KNO_3_ resulted in decreasing *S. apiospermum* counts at 18 °C as well as at 25 °C. With 4% KNO_3_ *S. apiospermum* proliferated. *S. dehoogii* numbers decrased a little at 18 °C with 2% KNO_3_ but still accounted for almost half of the *Scedosporium* population with 4% KNO_3_ at 18 °C. The numbers of *S. aurantiacum* rose with 2% KNO_3_ especially at 25 °C. The effect was also detectable at 18 °C. *S. boydii* was detected but played a minor role in this respect (Table 2 and Table 3).

Soil treatment with 5 and 10 g diesel/kg soil led to a strong increase in *S. dehoogii,* from 19.8 CFU/g (dw) to 421.9 and 653.5 CFU/g (dw) respectively (Table 2 and Table 3). In this case the higher diesel concentration as well as the higher temperature had a promoting effect. For *S. apiospermum,* a decrease in strain counts in the sample with 5 g diesel/kg at 18 °C, and slight increases with 5 g diesel/kg at 25 °C and 10 g diesel/kg at 18 °C were found. A more than five fold increase was counted with 10 g diesel/kg soil at 25 °C. *S. aurantiacum* and *S. boydii* were detected at the end of the survey but only in low numbers which were a little higher at 25 °C incubation temperature (Table 2 and Table 3). Isolated colony counts after three and six weeks as well as all raw data can be found as Supplementary Material.

## 4. Discussion

The opportunistic fungi arranged in the genus *Scedosporium* are known as significant opportunistic human pathogens, also due to the increasing number of immunocompromised patients [21]. Ways of infection can be subsumed as traumatic inoculation or aspiration of contaminated materials, mainly soil or soil suspensions. Colonization of CF patients lungs occur most probably via the inhalation of fungal particles possibly adhering to dust. For risk assessment regarding these fungi and the suitable description of infection routes, investigating possible reservoirs and factors enhancing the spread of *Scedosporium* spp. is strongly requested.

The present study shows, that the propagation of *Scedosporium* strains was strongest at elevated temperatures (25 °C) together with high concentrations of diesel. The largest proportion of the *Scedosporium* population was formed by *S. apiospermum* and *S. dehoogii*. Ulfig et al. [9,15] and April et al. [12,22] suggested that biodiesel was used as a C-source for biomass production, because the growth of *S. boydii* isolates was stimulated. In the case of diesel oil they observed a strain specific behaviour and a connection with the isolation source of the strains. Moreover, these findings are in line with the described genetic abilities of *S. apiospermum* which enable this species to degrade a number of different hydrocarbon sources [18]. Interestingly increased temperature has a positive effect on the *Scedosporium* populations in general, but also amplifies the effect of diesel or nitrate. Together with the observation of the *Scedosporium* population, the numbers of *Aspergillus* and *Penicillium* strains were also measured with snap samples (data not shown) and decreased over the course of the experiment. An explanation for the increase of *Scedosporium* numbers is that they are favored by higher temperatures and are able to use alkanes like diesel as carbon sources, which is probably poisonous for many other organisms. Therefore more space and nutrient resources can be exploited by the *Scedosporium* population. 

In urban soils, especially where adjacent surfaces are sealed, even in 15 cm depth can reach more than 30 °C, measured previously in Innsbruck/Austria (temperate climate, [23]). Opportunistic fungi are expected to survive high temperatures. *Scedosporium* strains are able to germinate at temperatures up to 40 °C and grow only at more than 12 °C [23]. Together with mineral oil contamination such habitats are therefore strongly influenced by two selective factors promoting *Scedosporium* populations. Moreover the high summer temperatures are followed by a period from October to April not reaching 12 °C, where *Scedosporium* spp. presumably are not able to grow but will survive. This is in accordance with our findings and the proven abundance in urban soils, especially near gas stations [8].

Also the higher concentration of nitrate was found to have an enhancing effect on the *Scedosporium* population growth. While less pronounced than the effect of diesel it was still significant at 25 °C. Kaltseis et al. [8] and Ulfig et al. [15] found a positive correlation between ammonium levels in soils and the abundance of *Scedosporium* strains. Conjointly with humic compounds, ammonium is the key source of nitrogen in soils. Furthermore, nitrogen entry in soils mainly comes from fertilization, grazing animals and atmospheric emission of nitrogen [24]. Following our results, factors like over-fertilization and animal husbandry together with globally and locally rising temperatures, may promote the abundance of *Scedosporium* in soils. 

The largest part of *Scedosporium* spp. increase at higher temperatures combined with nitrate and especially diesel was due to *S. apiospermum* and *S. dehoogii*. The other species and near relatives of epidemiologic importance were either not isolated at all (*S. desertorum*, *S. minutisporum*), or of minor importance for the increase of *Scedosporium* counts (*S. aurantiacum* and *S. boydii*). *Lomentospora prolificans*, which was formerly known as *S. prolificans* and is of clinical relevance, was neither isolated from the initial population, nor later. Nevertheless *S. aurantiacum* and *S. boydii* may increase under the described conditions and may represent a possible source of infection. *S. apiospermum* was found to be the most frequent *Scedosporium* species not only in our experiments but also in previous surveys [8,10,13] in soils. 

## 5. Conclusions

This work shows, that higher temperatures, mineral oil pollution and over-fertilization promote the distribution of opportunistic human pathogens from the genus *Scedosporium.* In short: pollution and high temperature periods support our pathogens. The findings from this work can also be helpful in predicting consequences of future challenges like climate change and environmental pollution. For more detailed descriptions of the influence of abiotic factors on *Scedosporium* growth, studies introducing steady state systems should be carried out in the future. Based on the presented data and further investigations *Scedosporium* strains could be established as bioremediation agents (i.e., [25]) and as indicator organisms for ecotoxicological, epidemiologic and public health features.

## Figures and Tables

**Table 1:** Fragment sizes (bp) after digestion with enzymes AluI, HaeIII, HinfI, RsaI, and Sau3AI for reference strains.

Taxa	AluI	HaeIII	HinfI	RsaI	Sau3AI
*Scedosporium apiospermum*	-	450	300	-	400
dH 20786		100	150		200
		50	150		
*Scedosporium aurantiacum*	240	450	300	-	400
dH 20791	160	100	150		200
	100	50	150		
	100				
*Scedosporium boydii*	400	450	300	-	400
dH 21103	200	100	150		200
		50	150		
*Scedosporium dehoogii*	400	350	300	-	400
dH 21018	200	100	150		200
		100	150		
		50			
*Lomentospora prolificans*	-	550	300	-	300
dH 20799		50	300		200
					100

**Table 2:** CFU values of *Scedosporium* species per gram soil (dw) after 0 and 9 weeks of incubation in (un)treated pots at 18 °C. A pooled sampling was used for the determination of CFU of week 0.

Treatment	no Treatment		2% KNO_3_	4% KNO_3_	5 g Diesel	10 g Diesel
Week	0	9	9	9	9	9
*S. apiospermum*	133.4	55.1	114.8	95.0	82.6	162.3
*S. dehoogii*	19.8	13.3	0.0	81.0	34.8	421.9
*S. aurantiacum*	5.7	13.3	38.3	0.0	11.6	0.0
*S. boydii*	0.0	13.3	0.0	0.0	0.0	64.9

**Table 3:** CFU values of *Scedosporium* species per gram soil (dw) after 0 and 9 weeks of incubation in (un)treated pots at 25 °C. A pooled sampling was used for the determination of CFU of week 0.

Treatment	No Treatment		2% KNO_3_	4% KNO_3_	5 g Diesel	10 g Diesel
Week	0	9	9	9	9	9
*S. apiospermum*	133.4	85.8	55.9	498.80	165.1	717.3
*S. dehoogii*	19.8	28.6	55.9	0.00	188.2	653.5
*S. aurantiacum*	5.7	28.6	190.8	81.20	15.4	111.6
*S. boydii*	0.0	0.0	26.3	0.0	15.4	111.6

## Data Availability

Original data can be found as Supplementary Materials.

## Supplementary Materials

The following supporting information can be downloaded at: https://www.mdpi.com/article/10.3390/jof9040403/s1, Table S1: Abundance of Scedosporium in soil samples at different incubation Temperatures; Table S2: Abundance of Scedosporium concerning different potassium nitrate concentrations (2% and 4% w/v) at 18 °C; Table S3: Abundance of Scedosporium concerning different potassium nitrate concentrations (2% and 4% w/v) at 25 °C; Table S4: Abundance of Scedosporium concerning different diesel concentrations (5 g and 10 g/kg soil) at 18 °C; Table S5: Abundance of Scedosporium concerning different diesel concentrations (5 g and 10 g/kg soil) at 25 °C; Table S6: Single values of CFU counted per plate, per milliliter soil suspension and per gram soil (dw) after 0 weeks of incubation of treated pots; Table S7: Single values of CFU counted per plate, per milliliter soil suspension and per gram soil (dw) after 3 weeks of incubation of treated pots; Table S8: Single values of CFU counted per plate, per milliliter soil suspension and per gram soil (dw) after 6 weeks of incubation of treated pots; Table S9: Single values of CFU counted per plate, per milliliter soil suspension and per gram soil (dw) after 9 weeks of incubation of treated pots.
