# Supplementary material for: Elevated Temperature, Nitrate and Diesel Oil Enhance the Distribution of the Opportunistic Pathogens Scedosporium spp
Source: J Fungi (Basel). 2023 Mar 24;9(4):403. doi: 10.3390/jof9040403 (PMC10144257; doi:10.3390/jof9040403)
Supplement: Supplementary file 1 [file jof-09-00403-s001.zip › jof-2270956-supplementary.pdf]

Supplementary Materials

Table S**1.** Abundance of *Scedosporium* in soil samples at different incubation temperatures. Values are given in CFU per gram soil (dw).

**Incubation temperature**	**Incubation period**	**Mean**	**Med**	**s**	**Min**	**Max**
**18 °C**	0 wk	177	143	75	95	333
	3 wk	241	244	58	190	321
	6 wk	202	190	79	95	333
	9 wk	95	95	44	48	143
**25 °C**	0 wk	156	125	113	48	333
	3 wk	219	214	72	143	333
	6 wk	256	238	98	95	429
	9 wk	143	143	62	95	238

Table S**2.** Abundance of *Scedosporium* concerning different potassium nitrate concentrations (2 % and 4 % w/v) at 18 °C. Values are given in CFU per gram soil (dw).

**KNO_3 (w/v)**	**Incubation period**	**Mean value**	**Median**	**Standard deviation**	**Minimum**	**Maximum**
**2%**	0 wk	143	149	83	48	286
	3 wk	295	304	105	143	429
	6 wk	125	95	59	71	214
	9 wk	153	143	102	36	381
**4%**	0 wk	149	125	69	71	250
	3 wk	201	238	72	48	250
	6 wk	112	107	68	48	238
	9 wk	176	179	33	143	238

Table S**3.** Abundance of *Scedosporium* concerning different potassium nitrate concentrations (2 % and 4 % w/v) at 25 °C. Values are given in CFU per gram soil (dw).

**KNO_3 (w/v)**	**Incubation period**	**Mean value**	**Median**	**Standard deviation**	**Minimum**	**Maximum**
**2%**	0 wk	147	143	87	36	333
	3 wk	372	357	133	238	571
	6 wk	265	250	84	143	393
	9 wk	329	327	50	238	393
**4%**	0 wk	134	143	54	48	190
	3 wk	241	238	111	107	333
	6 wk	448	446	81	286	571

9 wk	580	589	71	524	667

Table S**4.** Abundance of *Scedosporium/Pseudallescheria* concerning different Diesel concentrations (5 g and 10 g/kg soil) at 18 °C. Values are given in CFU per gram soil (dw).

**Diesel g/kg soil**	**Incubation period**	**Mean value**	**Median**	**Standard deviation**	**Minimum**	**Maximum**
**5 g**	0 wk	202	214	76	95	286
	3 wk	318	304	88	238	476
	6 wk	167	167	109	48	381
	9 wk	129	125	83	35	285
**10 g**	0 wk	106	107	78	0	214
	3 wk	313	327	35	250	357
	6 wk	682	643	92	571	857
	9 wk	649	619	126	523	892

Table S**5.** Abundance of *Scedosporium/Pseudallescheria* concerning different Diesel concentrations (5 g and 10 g/kg soil) at 25 °C. Values are given in CFU per gram soil (dw).

**Diesel g/kg soil**	**Incubation period**	**Mean value**	**Median**	**Standard deviation**	**Minimum**	**Maximum**
**5 g**	0 wk	196	167	96	107	381
	3 wk	183	190	81	95	321
	6 wk	237	232	101	107	429
	9 wk	384	375	88	286	524
**10 g**	0 wk	159	196	103	36	286
	3 wk	317	304	116	190	357
	6 wk	1129	1119	223	893	1429
	9 wk	1594	1554	189	1357	1810

Single values (CFU) of the abundance of *Scedosporium*

Table S6. Single values of CFU counted per plate, per millilitre soil suspension and per gram soil (dw) after 0 weeks of incubation of treated pots (**Sampling I**). Concerning parallels 1 to 4, 300 µL soil suspension (1:10) were plated. On parallels 5 to 8, 400 µL soil suspension (1:10) were plated.

	Parallels	CFU/plate 1:10	CFU/mL soilsuspension	CFU/g soil (dw)	Mean	Median	Standard deviation
**Pot 1**	1	7	23,33	333,33			
**18 °C**	2	5	16,67	238,10			
	2	3	10,00	142,86			
	4	2	6,67	95,24			
	5	4	10,00	142,86			
	6	5	12,50	178,57			
	7	4	10,00	142,86			
	8	4	10,00	142,86	177,08	142,86	75,14
**Pot 2**	1	1	3,33	47,62			
**18 °C**	2	6	20,00	285,71			
**2 % KNO_3**	3	3	10,00	142,86			
	4	5	16,67	238,10			
	5	4	10,00	142,86			
	6	2	5,00	71,43			
	7	2	5,00	71,43			
	8	4	10,00	142,86	142,86	142,86	83,45
**Pot 3**	1	4	13,33	190,48			
**18 °C**	2	2	6,67	95,24			
**4 % KNO_3**	3	2	6,67	95,24			
	4	5	16,67	238,10			
	5	3	7,50	107,14			
	6	7	17,50	250,00			
	7	4	10,00	142,86			
	8	2	5,00	71,43	177,08	160,71	86,89
**Pot 4**	1	2	6,67	95,24			
**18 °C**	2	6	20,00	285,71			
**5 g Diesel**	3	6	20,00	285,71			
	4	2	6,67	95,24			
	5	5	12,50	178,57			
	6	7	17,50	250,00			
	7	6	15,00	214,29			
	8	6	15,00	214,29	197,92	214,29	78,18
**Pot 5**	1	3	10,00	142,86			
**18 °C**	2	1	3,33	47,62			
**10 g Diesel**	3	3	10,00	142,86			

	4	4	13,33	190,48			
	5	2	5,00	71,43			
	6	1	2,50	35,71			
	7	6	15,00	214,29			
	8	0	0,00	0,00	102,68	95,24	79,72
**Pot 6**	1	1	3,33	47,62			
**25 °C**	2	7	23,33	333,33			
	3	1	3,33	47,62			
	4	6	20,00	285,71			
	5	2	5,00	71,43			
	6	6	15,00	214,29			
	7	5	12,50	178,57			
	8	2	5,00	71,43	156,25	125,00	113,37
**Pot 7**	1	7	23,33	333,33			
**25 °C**	2	2	6,67	95,24			
**2 % KNO_3**	3	3	10,00	142,86			
	4	3	10,00	142,86			
	5	4	10,00	142,86			
	6	1	2,50	35,71			
	7	5	12,50	178,57			
	8	3	7,50	107,14	147,32	142,86	86,54
**Pot 8**	1	4	13,33	190,48			
**25 °C**	2	1	3,33	47,62			
**4 % KNO_3**	3	3	10,00	142,86			
	4	4	13,33	190,48			
	5	2	5,00	71,43			
	6	5	12,50	178,57			
	7	3	7,50	107,14			
	8	4	10,00	142,86	133,93	142,86	54,28
**Pot 9**	1	3	10,00	142,86			
**25 °C**	2	8	26,67	380,95			
**5 Diesel**	3	6	20,00	285,71			
	4	4	13,33	190,48			
	5	3	7,50	107,14			
	6	3	7,50	107,14			
	7	4	10,00	142,86			
	8	6	15,00	214,29	196,43	166,67	95,66
**Pot 10**	1	6	20,00	285,71			
**25 °C**	2	5	16,67	238,10			
**10 Diesel**	3	5	16,67	238,10			
	4	1	3,33	47,62			
	5	6	15,00	214,29			
	6	1	2,50	35,71			
	7	5	12,50	178,57			

	8	1	2,50	35,71	159,23	196,43	103,38

Table S**7.** Single values of CFU counted per plate, per millilitre soil suspension and per gram soil (dw) after 3 weeks of incubation of treated pots (**Sampling II**). Concerning parallels 1 to 4, 300 µL soil suspension (1:10) were plated. On parallels 5 to 8, 400 µL soil suspension (1:10) were plated.

	Parallels	CFU/plate 1:10	CFU/mL soilsuspension	CFU/g soil (dw)	Mean	Median	Standard deviation
**Pot 1**	1	4	13,33	190,48			
**18 °C**	2	5	16,67	238,10			
	2	4	13,33	190,48			
	4	6	20,00	285,71			
	5	6	15,00	214,29			
	6	7	17,50	250,00			
	7	4	10,00	142,86			
	8	9	22,50	321,43	229,17	226,19	57,18
**Pot 2**	1	3	10,00	142,86			
**18 °C**	2	5	16,67	238,10			
**2 % KNO_3**	3	9	30,00	428,57			
	4	7	23,33	333,33			
	5	9	22,50	321,43			
	6	8	20,00	285,71			
	7	5	12,50	178,57			
	8	12	30,00	428,57	260,42	261,90	135,65
**Pot 3**	1	1	3,33	47,62			
**18 °C**	2	5	16,67	238,10			
**4 % KNO_3**	3	5	16,67	238,10			
	4	5	16,67	238,10			
	5	7	17,50	250,00			
	6	5	12,50	178,57			
	7	6	15,00	214,29			
	8	0	0,00	0,00	200,68	238,10	71,57
**Pot 4**	1	10	33,33	476,19			
**18 °C**	2	5	16,67	238,10			
**5 g Diesel**	3	8	26,67	380,95			
	4	5	16,67	238,10			
	5	10	25,00	357,14			
	6	10	25,00	357,14			
	7	7	17,50	250,00			
	8	7	17,50	250,00	318,45	303,57	87,89
**Pot 5**	1	7	23,33	333,33			
**18 °C**	2	7	23,33	333,33			
**10 g Diesel**	3	6	20,00	285,71			
	4	7	23,33	333,33			

	5	9	22,50	321,43			
	6	7	17,50	250,00			
	7	8	20,00	285,71			
	8	10	25,00	357,14	312,50	327,38	35,29
**Pot 6**	1	3	10,00	142,86			
**25 °C**	2	5	16,67	238,10			
	3	9	30,00	428,57			
	4	0	0,00	0,00			
	5	8	20,00	285,71			
	6	5	12,50	178,57			
	7	6	15,00	214,29			
	8	4	10,00	142,86	232,99	214,29	100,51
**Pot 7**	1	12	40,00	571,43			
**25 °C**	2	5	16,67	238,10			
**2 % KNO_3**	3	11	36,67	523,81			
	4	9	30,00	428,57			
	5	7	17,50	250,00			
	6	8	20,00	285,71			
	7	12	30,00	428,57			
	8	7	17,50	250,00	372,02	357,14	133,29
**Pot 8**	1	7	23,33	333,33			
**25 °C**	2	9	30,00	428,57			
**4 % KNO_3**	3	4	13,33	190,48			
	4	5	16,67	238,10			
	5	7	17,50	250,00			
	6	4	10,00	142,86			
	7	3	7,50	107,14			
	8	grown over	-	-	241,50	238,10	110,98
**Pot 9**	1	4	13,33	190,48			
**25 °C**	2	2	6,67	95,24			
**5 Diesel**	3	2	6,67	95,24			
	4	4	13,33	190,48			
	5	3	7,50	107,14			
	6	6	15,00	214,29			
	7	7	17,50	250,00			
	8	9	22,50	321,43	183,04	190,48	80,98
**Pot 10**	1	6	20,00	285,71			
**25 °C**	2	5	16,67	238,10			
**10 Diesel**	3	12	40,00	571,43			
	4	4	13,33	190,48			
	5	7	17,50	250,00			
	6	9	22,50	321,43			
	7	9	22,50	321,43			
	8	10	25,00	357,14	316,96	303,57	115,94

Table S**8.** Single values of CFU counted per plate, per millilitre soil suspension and per gram soil (dw) after 6 weeks of incubation of treated pots (**Sampling III**). Concerning parallels 1 to 4, 300 µL soil suspension (1:10) were plated. On parallels 5 to 8, 400 µL soil suspension (1:10) were plated.

	Parallels	CFU/plate 1:10	CFU/mL soilsuspension	CFU/g soil (dw)	Mean	Median	Standard deviation
**Pot 1**	1	4	13,33	190,48			
**18 °C**	2	5	16,67	238,10			
	2	2	6,67	95,24			
	4	3	10,00	142,86			
	5	4	13,33	190,48			
	6	3	10,00	142,86			
	7	7	23,33	333,33			
	8	6	20,00	285,71	202,38	190,48	79,48
**Pot 2**	1	2	6,67	95,24			
**18 °C**	2	3	10,00	142,86			
**2 % KNO_3**	3	2	6,67	95,24			
	4	2	6,67	95,24			
	5	2	5,00	71,43			
	6	2	5,00	71,43			
	7	6	15,00	214,29			
	8	6	15,00	214,29	125,00	95,24	59,35
**Pot 3**	1	1	3,33	47,62			
**18 °C**	2	5	16,67	238,10			
**4 % KNO_3**	3	3	10,00	142,86			
	4	3	10,00	142,86			
	5	2	5,00	71,43			
	6	4	10,00	142,86			
	7	1	2,50	35,71			
	8	2	5,00	71,43	111,61	107,14	67,62
**Pot 4**	1	4	13,33	190,48			
**18 °C**	2	3	10,00	142,86			
**5 g Diesel**	3	1	3,33	47,62			
	4	8	26,67	380,95			
	5	6	15,00	214,29			
	6	6	15,00	214,29			
	7	2	5,00	71,43			
	8	2	5,00	71,43	166,67	166,67	109,48
**Pot 5**	1	15	50,00	714,29			
**18 °C**	2	13	43,33	619,05			
**10 g Diesel**	3	16	53,33	761,90			
	4	18	60,00	857,14			

	5	16	40,00	571,43			
	6	18	45,00	642,86			
	7	18	45,00	642,86			
	8	18	45,00	642,86	681,55	642,86	91,72
**Pot 6**	1	4	13,33	190,48			
**25 °C**	2	5	16,67	238,10			
	3	6	20,00	285,71			
	4	2	6,67	95,24			
	5	5	16,67	238,10			
	6	9	30,00	428,57			
	7	7	23,33	333,33			
	8	5	16,67	238,10	255,95	238,10	98,38
**Pot 7**	1	5	16,67	238,10			
**25 °C**	2	3	10,00	142,86			
**2 % KNO_3**	3	7	23,33	333,33			
	4	7	23,33	333,33			
	5	7	17,50	250,00			
	6	5	12,50	178,57			
	7	11	27,50	392,86			
	8	7	17,50	250,00	264,88	250,00	83,88
**Pot 8**	1	10	33,33	476,19			
**25 °C**	2	12	40,00	571,43			
**4 % KNO_3**	3	9	30,00	428,57			
	4	9	30,00	428,57			
	5	13	32,50	464,29			
	6	12	30,00	428,57			
	7	14	35,00	500,00			
	8	8	20,00	285,71	447,92	446,43	81,48
**Pot 9**	1	3	10,00	142,86			
**25 °C**	2	9	30,00	428,57			
**5 Diesel**	3	6	20,00	285,71			
	4	6	20,00	285,71			
	5	7	17,50	250,00			
	6	6	15,00	214,29			
	7	5	12,50	178,57			
	8	3	7,50	107,14	236,61	232,14	100,90
**Pot 10**	1	19	63,33	904,76			
**25 °C**	2	24	80,00	1142,86			
**10 Diesel**	3	27	90,00	1285,71			
	4	23	76,67	1095,24			
	5	39	97,50	1392,86			
	6	40	100,00	1428,57			
	7	25	62,50	892,86			
	8	25	62,50	892,86	1129,46	1119,0	222,67

Table S**9.** Single values of CFU counted per plate, per millilitre soil suspension and per gram soil (dw) after 9 weeks of incubation of treated pots (**Sampling IV**). Concerning parallels 1 to 4, 300 µL soil suspension (1:10) were plated. On parallels 5 to 8, 400 µL soil suspension (1:10) were plated.

	Parallels	CFU/plate 1:10	CFU/mL soilsuspension	CFU/g soil (dw)	Mean	Median	Standard deviation
**Pot 1**	1	1	3,33	47,62			
**18 °C**	2	1	3,33	47,62			
	2	3	10,00	142,86			
	4	3	10,00	142,86			
	5	2	6,67	95,24			
	6	3	10,00	142,86			
	7	1	3,33	47,62			
	8	2	6,67	95,24	95,24	95,24	44,09
**Pot 2**	1	8	26,67	380,95			
**18 °C**	2	3	10,00	142,86			
**2 % KNO_3**	3	3	10,00	142,86			
	4	2	6,67	95,24			
	5	3	7,50	107,14			
	6	5	12,50	178,57			
	7	4	10,00	142,86			
	8	1	2,50	35,71	153,27	142,86	101,50
**Pot 3**	1	5	16,67	238,10			
**18 °C**	2	4	13,33	190,48			
**4 % KNO_3**	3	3	10,00	142,86			
	4	4	13,33	190,48			
	5	4	10,00	142,86			
	6	5	12,50	178,57			
	7	4	10,00	142,86			
	8	5	12,50	178,57	175,60	178,57	32,91
**Pot 4**	1	2	6,67	95,24			
**18 °C**	2	4	13,33	190,48			
**5 g Diesel**	3	3	10,00	142,86			
	4	6	20,00	285,71			
	5	1	2,50	35,71			
	6	4	10,00	142,86			
	7	3	7,50	107,14			
	8	1	2,50	35,71	129,46	125,00	82,59
**Pot 5**	1	11	36,67	523,81			
**18 °C**	2	12	40,00	571,43			
**10 g Diesel**	3	14	46,67	666,67			
	4	12	40,00	571,43			
	5	15	37,50	535,71			

	6	19	47,50	678,57			
	7	21	52,50	750,00			
	8	25	62,50	892,86	648,81	619,05	126,15
**Pot 6**	1	4	13,33	190,48			
**25 °C**	2	2	6,67	95,24			
	3	3	10,00	142,86			
	4	3	10,00	142,86			
	5	2	6,67	95,24			
	6	4	13,33	190,48			
	7	5	16,67	238,10			
	8	1	3,33	47,62	142,86	142,86	62,35
**Pot 7**	1	8	26,67	380,95			
**25 °C**	2	7	23,33	333,33			
**2 % KNO_3**	3	6	20,00	285,71			
	4	5	16,67	238,10			
	5	9	22,50	321,43			
	6	11	27,50	392,86			
	7	9	22,50	321,43			
	8	10	25,00	357,14	328,87	327,38	50,48
**Pot 8**	1	11	36,67	523,81			
**25 °C**	2	12	40,00	571,43			
**4 % KNO_3**	3	14	46,67	666,67			
	4	14	46,67	666,67			
	5	13	32,50	464,29			
	6	17	42,50	607,14			
	7	17	42,50	607,14			
	8	15	37,50	535,71	580,36	589,29	70,79
**Pot 9**	1	11	36,67	523,81			
**25 °C**	2	9	30,00	428,57			
**5 Diesel**	3	10	33,33	476,19			
	4	6	20,00	285,71			
	5	9	22,50	321,43			
	6	10	25,00	357,14			
	7	8	20,00	285,71			
	8	11	27,50	392,86	383,93	375,00	87,89
**Pot 10**	1	38	126,67	1809,52			
**25 °C**	2	33	110,00	1571,43			
**10 Diesel**	3	32	106,67	1523,81			
	4	38	126,67	1809,52			
	5	43	107,50	1535,71			
	6	50	125,00	1785,71			
	7	38	95,00	1357,14			
	8	38	95,00	1357,14	1593,75	1553,57	189,24
